# Virtual reality-based intervention to improve pre-operative anxiety in patients undergoing planned oocyte cryopreservation: a randomized clinical trial

**DOI:** 10.1007/s10815-025-03571-w

**Published:** 2025-07-09

**Authors:** Yael Suissa-Cohen, Rachelle Frenkel, Lea Nevo, Hadas Allouche Kam, Yaakov Bentov, Mary Godin, Efrat Esh Broder, Ranit Hizkiyahu, Chana Adler Lazarovits, Ofer Beharier, Anat Hershko Klement

**Affiliations:** 1 Department of Obstetrics and Gynecology, IVF Unit, Hadassah Hospital Mount Scopus, Jerusalem, Israel; 2 Department of Obstetrics and Gynecology, Faculty of Medicine, Hebrew University of Jerusalem, Jerusalem, Israel

**Keywords:** Oocyte retrieval, Planned oocyte cryopreservation, Virtual reality; Anxiety, Visual analogue scale

## Abstract

**Purpose:**

To investigate the impact of virtual reality (VR) exposure on anxiety levels in patients undergoing planned oocyte cryopreservation.

**Methods:**

Participants were randomized into a routine management group or a VR group. The VR group underwent a 20-min VR session featuring scenic movies before entering the operating room. All procedures were conducted under anesthesia. Demographic data collected included age, BMI, cycle number, expected and retrieved eggs, and whether patients were accompanied. The primary outcome was anxiety change, measured using the State-Trait Anxiety Inventory (STAI). A sample size of 12 patients per group was calculated to detect a 5-point reduction in STAI scores. Secondary measures included anxiety-related vital signs and visual analogue scale (VAS, 1–10). Data were collected at admission, pre-procedure, and post-recovery.

**Results:**

Of the 30 patients recruited, 16 were assigned to routine management and 14 to the VR group. Baseline anxiety scores (S-STAI) of both groups were low and almost identical (VR group 24.8 ± 10.2, Routine management group 24.75 ± 11.1, *p* = 0.897). The VR group showed a significant reduction in STAI scores from admission to pre-procedure, while the routine group experienced an increase (*p* = 0.02). Systolic blood pressure decreased more in the VR group post-recovery (− 9.7 ± 12.3 mmHg vs. − 1.3 ± 13.1 mmHg, *p* = 0.08), although not reaching a statistical significance. VAS scores also showed a greater reduction in the VR group from admission to pre-procedure (*p* = 0.04).

**Conclusion:**

VR exposure before oocyte retrieval significantly reduced anxiety, as indicated by STAI scores, systolic blood pressure, and VAS scores.

**Trial registration:**

Clinical-Trials NCT06280222, Registered online 07.12.2023.

## Introduction

Planned oocyte cryopreservation has gained popularity in recent years [1, 2]. The awareness of age-related fertility decline, alongside constraints of the modern world and employment demands, leads many women to postpone childbearing. A well-established option for these women is planned oocyte cryopreservation, which offers a potential opportunity for genetic motherhood at older ages [3]. With the fast distribution and improved accessibility to this service, many women worldwide elect to cryo-preserve their eggs.

Although patients going through planned oocyte cryopreservation were not diagnosed with infertility, they demonstrate a compromised quality of life, similar to infertile patients [4]. Both IVF and oocyte cryopreservation patients were reported to express stress during treatment. There were no significant differences in reported anxiety between sub-fertile and planned oocyte cryopreservation patients. The anxiety and stress could be attributed to the general anesthesia, the procedure outcomes, or the need for follow-up procedures, as well as general life circumstances [5].

The use of virtual reality (VR) in medicine has gained popularity in recent years. This modality was found to be effective in reducing pain and analgesia use, as well as anxiety, in pediatric and adult patients, including during labor and childbirth, with negligible side effects [6–8]. It was also proposed as a method to relieve pain and psychological symptoms before and during hysterosalpingography and outpatient hysteroscopy [9, 10].

The role of VR in reproductive medicine was also evaluated in several clinical trials. This modality for reducing anxiety in IVF cycles, specifically during embryo transfer, was tested as a means to reduce and as a way to minimize discomfort in selected patients [11, 12]. However, its effectiveness appears to vary depending on the patient population, procedure type, and assessment methods. The use of virtual reality (VR) during hysterosalpingography (HSG) does not appear to reduce procedural pain scores compared to HSG without VR. In a randomized trial, patients undergoing HSG as part of an infertility work-up were assigned to either HSG with or without VR. The authors concluded that VR did not provide any significant improvement in pain or anxiety [13]. This finding contrasts with reports on VR use during office hysteroscopy. In a separate randomized trial evaluating VR as a distraction technique for managing acute pain and anxiety during outpatient hysteroscopy, VR was associated with significant pain and anxiety reduction. However, the study population primarily consisted of menopausal patients rather than infertile patients [10].

In another preliminary randomized study, VR exposure was administered before embryo transfer (ET) and compared to routine care. Anxiety was assessed at three time points: recruitment, pre-ET, and post-ET using the State-Trait Anxiety Inventory (STAI) questionnaire, as well as heart rate (HR) and blood pressure (BP) measurements. Anxiety scores were comparable between the VR and control groups, and clinical pregnancy rates did not differ significantly [11]. Notably, anxiety assessment began at recruitment, prior to treatment initiation, which may have influenced response patterns.

A separate randomized controlled trial investigated VR exposure before oocyte retrieval in sub-fertile patients. Anxiety was measured using both the visual analog scale (VAS) and the STAI questionnaire. The first evaluation occurred after intravenous line insertion (baseline), the second immediately following the VR session, and the final assessment after patients were informed of the number of oocytes retrieved. VR significantly reduced both preoperative and pre-discharge anxiety according to VAS and STAI scores. However, the reference group in this study consisted of patients receiving combined VR and hypnosis, which produced similar anxiety reduction trends and magnitudes [14].

Given the variability in findings across different reproductive procedures, further research is needed to better understand the role of VR in anxiety and pain management. Elective oocyte preservation, in particular, represents an area for investigation in this essence, as patients undergoing this procedure often experience high levels of anticipatory anxiety [15]. Exploring VR’s potential to alleviate anxiety in this population could improve the overall patient experience and procedural comfort. To our knowledge, no clinical studies have addressed VR as a means of reducing stress or possibly improving the success rate during planned oocyte cryopreservation. This study focused on stress reduction among patients undergoing oocyte retrieval for cryopreservation from choice. The aim of the study was to assess the effectiveness of VR in reducing stress among a selected population seeking oocyte cryopreservation for elective reasons, without a diagnosis of subfertility.

## Materials and methods

This randomized controlled trial was conducted in a single university-affiliated IVF clinic (Hadassah Hospital Mount Scopus, Jerusalem, Israel) from November 2023 to February 2024. The study was approved by the Hadassah research ethics board (0497–23-HMO REB) and was registered in ClinicalTrials.gov registry (NCT06280222) on December 07, 2023.

**Fig. 1:**
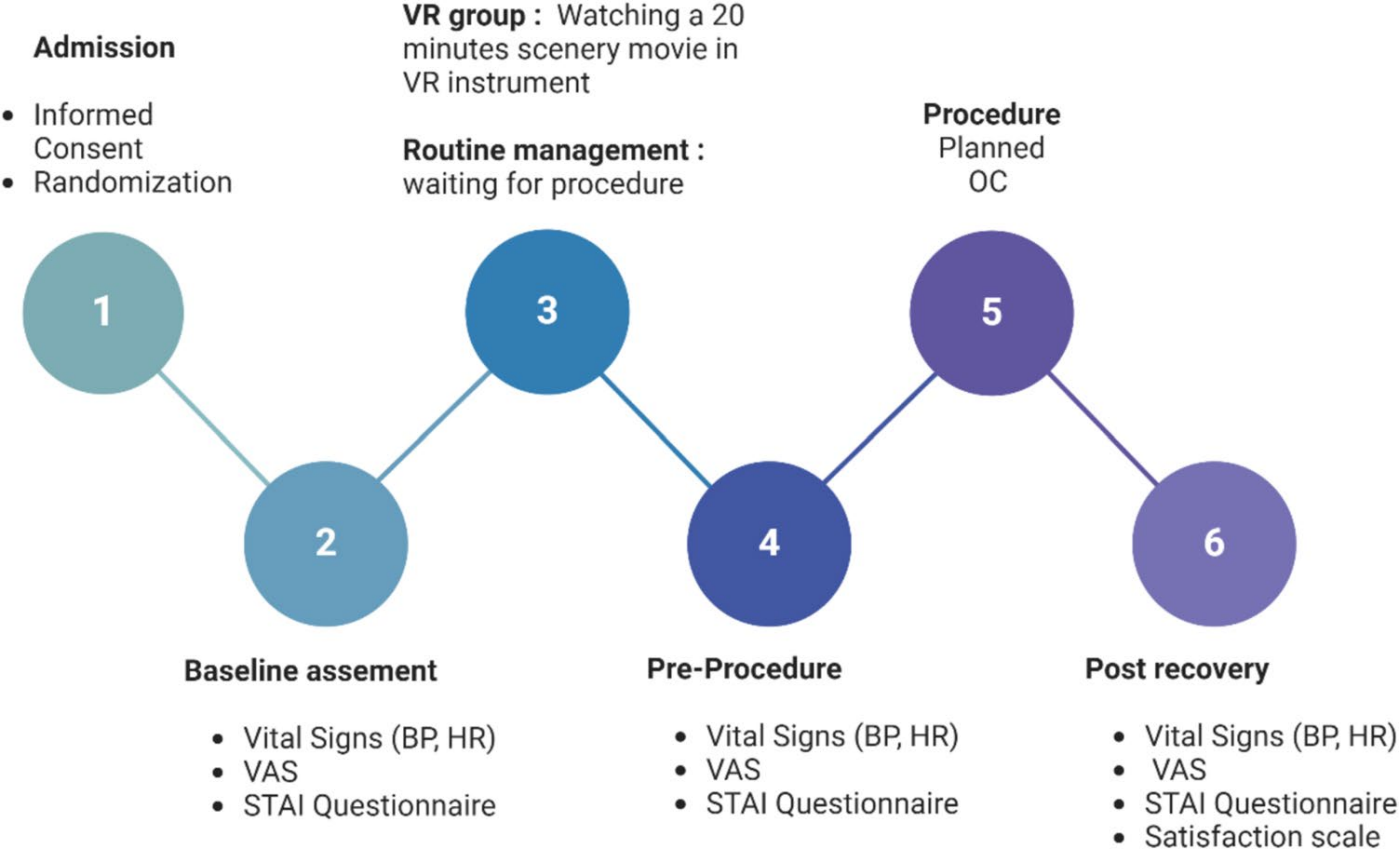
Study time-line

The primary outcome measure was the State Trait Anxiety Inventory (STAI) score, a validated tool to assess anxiety in a clinical setting [16, 17]. This questionnaire measures two types of anxiety—state anxiety and trait anxiety. STAI consists of two separate 20-item scales: one assessing state anxiety (S-STAI), referring to how a person is feeling at the very time of a perceived threat, and the other assessing trait anxiety (T-STAI) referring to a general perception. Each item is rated on a 4-point scale, ranging from “not at all” to “very much so” for state anxiety, and from “almost never” to “almost always” for trait anxiety. A score of up to 30 represents low anxiety, 31–45 indicates moderate anxiety, and a score > 45 is classified as high anxiety. This tool is frequently employed in both clinical and research settings to assess the psychological impact of medical procedures or stressors. Additional anxiety measures were the visual analog scale (VAS) for anxiety score [5], blood pressure (BP), and heart rate (HR): The VAS is a simple, self-report tool commonly used to assess subjective experiences, such as pain or anxiety, along a continuous line. The scale ranges from 0 (no anxiety) to 10 (extreme anxiety), and participants mark a point on the line that represents their current level of anxiety. This scale is effective in capturing changes in anxiety levels during and after interventions [18]. All data were collected at three time points: admission, pre-operative (after watching the VR video, before entering the procedure room), and before discharge (Fig. 1).

Eligible patients were offered to take part upon admission to the surgical outpatient clinic on the morning of oocyte retrieval. Only patients undergoing planned oocyte retrieval were approached. Patients were consented by a trained research nurse (surgical outpatient clinic), who was not involved in the prior clinical care, nor with the randomization process. The nurses were responsible for monitoring and documenting vital signs in the research file, as well as administering the questionnaires throughout the process. At each point of care, the patients were provided with the questionnaire to complete independently in the privacy of their bed.

Exclusion criteria were use of any medications for pain relief/anti-anxiety/anti-depressants, risk for seizures or previously diagnosed seizure disorder, sensitivity to flashing light/motion, a known predisposition to nausea/dizziness (vertigo, etc.), and any injury to the eyes/face/neck that would limit the use of the hardware, including blindness. In addition, patients undergoing fertility preservation for medical reasons or gender-affirming care were excluded.

In our institution, oocyte retrieval procedures are performed during the morning hours; all are conducted under anesthesia. Patients are asked to arrive at least 2 h before the surgical procedure. Patients wait in a private bed surrounded by curtains and are routinely monitored for blood pressure and pulse rate before the procedure. All patients are supervised routinely by nurses. One chaperon is allowed to accompany the patient until the procedure.

Upon signing the informed consent, patients were randomized into two groups: routine management (waiting for the procedure with no intervention) or exposure to a scenery video through a VR session. Staff members were not blinded to treatment allocation. The block randomization was performed in the gynecology outpatient clinic, removed from the IVF unit.

All patients in the intervention group watched three identical consecutive scenery movies (Virtual Nature 360°—Nature Meditation for VR Quest, Four Seasons. Winter Forest. Relax Flight. 360° and 360° VR—Morning Walk in Nature—4k Virtual Reality, Norwegian). Total session duration for the intervention group was 20 min.

The VR instrument utilized was an Oculus Quest 2 (Instructions for using the Oculus Quest 2 headset—VARWIN DOCS), which was donated to the Obstetrics and Gynecology department in Hadassah Medical Center. The VR was used as a viewer (Virtual Nature 360 for Oculus Quest). Each instrument was cleaned following use with special anti-septic wipes.

The intervention was immediately discontinued when a patient requested to discontinue watching, if discomfort or a side effect was reported, or when 20 min had elapsed.

The following demographic parameters were collected into an electronic database: age, AMH level, body mass index (BMI), cycle number, number of eggs retrieved, and whether lower than expected according to the last monitoring visit before retrieval, as well as whether accompanied by a relative/friend/parents or arrived alone. In addition, before discharge, each patient was asked to grade her general satisfaction with the procedure on a 1–10 scale, which was also recorded.

### Sample size calculation

The sample size calculation was based on previous studies indicating high anxiety levels among fertility patients undergoing procedures such as oocyte retrieval (77.4 ± 16.6) or embryo transfer (76.5 ± 18) [5]. To demonstrate a 5-point reduction in the STAI score with 80% power and *α* = 0.05, a sample size of 12 patients was required in each group. The study aimed to recruit 30 patients to account for potential dropouts before reaching the necessary sample size.

### Statistical analysis

Each stress score was compared for each patient using difference calculation between two assessment points, hence the change in each patient’s anxiety level between two assessment points. For example, “pre-procedure VAS minus baseline VAS,” “pre-discharge STAI score minus baseline STAI score.”

These difference scores were then used to obtain mean difference values for each group. To compare these mean difference values between the two independent groups (routine management vs. VR), appropriate statistical tests were applied based on data distribution:

A non-paired *t*-test was used for normally distributed variables, Mann–Whitney *U* test for non-parametric variables, and chi-squared for categorical variables.

## Results

**Fig. 2:**
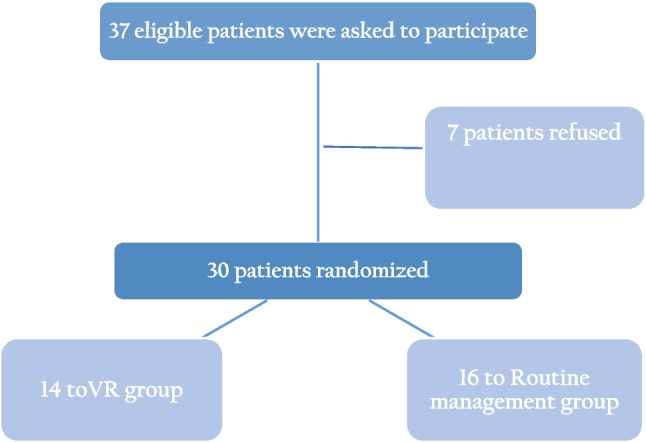
Group allocation and randomization

A total of 30 patients were recruited to the study; 14 were randomized to the routine management group and 12 to the VR group (randomization ratio 7:6) (Fig. 2). None of the patients had a diagnosed chronic medical condition. A total of seven women declined participation due to personal preference (Fig. 2). No safety events were reported while watching the VR movie, and none of the participants asked to stop the session. One patient reported slight dizziness after completing watching the movies.

There were no significant demographic differences between the study groups (Table 1). In the VR group, four (28%) patients arrived alone, in contrast to the routine management group in which all 14 were accompanied by a chaperon (*p* = 0.04). Importantly, the basic stationary anxiety scores (S-STAI) of both groups were almost identical (VR group 24.8 ± 10.2 and the routine management group 24.75 ± 11.1, *p* = 0.897). Before entering the procedure room, S-STAI score was 21.14 ± 8.3 in the VR group versus 28.44 ± 14.6 in the routine management group (differences’ analysis is detailed in the following paragraph) and upon discharge 18.6 ± 9.7 and 19.0 ± 9.0 respectively.

**Table 1:** Patient characteristics according to study group

Variable*	Virtual reality (*n* = 14)	Routine management (*n* = 16)	*p*-value
Age (years)	35.0 ± 2.5	33.4 ± 2.3	0.09
AMH (ng/ml)	1.4	2.0	0.25
Body mass index	21.7 ± 3	22.8 ± 2.8	0.32
Cycle number	1.75 ± 0.8	1.64 ± 1.3	0.79
Number of retrieved eggs	11.6 ± 7	13.1 ± 8.5	0.61
Patients with fewer eggs than expected	*n* = 4 (28.6)	*n* = 1 (6.3)	0.16
Patients without a chaperone	*n* = 4 (28.6)	0	0.04

^*^Data are presented as *n* (%) or mean ± standard deviation

We tracked a statistically significant decrease (*p* = 0.02) in the S-STAI of the VR group from admission to pre-procedure, as expressed by the S-STAI (Table 2). The VR group demonstrated a decrease, while the scores of the routine management group increased (Table 2).

**Table 2:** Calculated differences in anxiety scores by study group and point of care

	*Pre-Procedure vs. Admission*									*After recovery vs. Admission*										
	*Pulse*		*Systolic BP*		*STAI State*		*STAI trait*		*VAS*	*Pulse*			*Systolic BP*		*STAI State*		*STAI trait*		*VAS*	
	Mean ± SD	*P*-Value	Mean ± SD	*P*-Value	Mean ± SD	*P*-Value	Mean ± SD	*P*-Value	Mean ± SD	*P*-Value	Mean ± SD	*P*-Value	Mean ± SD	*P*-Value	Mean ± SD	*P*-Value	Mean ± SD	*P*-Value	Mean ± SD	*P*-Value
Virtual Reality (*n* = 14)	0.0± 5.7	0.38	5.65± 12	0.23	–3.7 ± 8.6	*0.02	–1 ± 1.66	0.89	–0.29 ± 1.54	*0.03	–6.4 ± 10.5	0.75	–9.7 ± 12.3	0.08	–6.2 ± 7.7	0.87	–6.2 ± 7.7	0.51	–1.78 ± 3.51	0.78
Routine management (*n* = 16)	–2.3 ± 8		0.56 ± 10.6		3.7 ± 7.6		–1.12 ± 3.2		0.87 ± 1.40		–4.8 ± 15.4		–1.3± 13.1		–5.7 ± 8.4		–2.06 ± 2.6		–2.31 ± 2.38	

The proportion of patients exhibiting moderate to severe anxiety, as measured by an S-STAI > 30, followed a similar trend; baseline levels of moderate to severe anxiety did not differ significantly between groups (*p* = 0.4). After the intervention and before the procedure, a significant group difference was observed: 8.3% of participants in the VR group expressed moderate to severe anxiety, compared to 50% in the routine management group (*p* = 0.04) (Table 3).

**Table 3:** Proportion of patients exhibiting moderate-severe S-STAI score at different points of care

	**VR group**	**Routine management**	*p*-value*
Admission	4/12 (33.3%)	3/14 (21.4%)	0.4
Pre-operative	1/12 (8.3%)	7/14 (50.0%)	**0.04**
Discharge	1/12 (8.3%)	1/14 (7.1%)	> 0.5

^*^Fisher’s *P*

Baseline VAS scores were comparable between study groups: 3.3 ± 2.5 in the VR group versus 3.2 ± 2.4 in the routine management (*p* = 0.9). Pre-procedure scores were 3.0 ± 2.5 (VR) and 4.1 ± 2.9 (routine), and pre-discharge values were 1.2 ± 2.2 and 1.8 ± 1.9, respectively. VAS differences were significantly in favor of stress reduction in the VR group for the pre-procedure versus admission values, though the magnitude was low: a + 0.8 ± 1.4 increase in the routine management group versus a decrease of − 0.3 ± 1.5 in the VR group (*p* = 0.04).

Decreased anxiety among the VR group was also expressed in the systolic blood pressure, which decreased by − 9.7 ± 12.3 mmHg in the VR group after recovery (Table 2, *p* = 0.08), as compared to − 1.3 ± 13.1 in the routine management group, though not reaching a statistical significance.

Satisfaction scores (1–10) were generally high and not different when between study groups (routine management 8.4 ± 1.4 versus VR 8.2 ± 1.8, *p* = 0.74).

## Discussion

This study reports an improvement in stress parameters after observing VR nature films among patients who were undergoing planned oocyte cryopreservation. We detected a significant decrease in pre-procedure VAS anxiety scores and S-STAI anxiety levels following a VR session, as compared to admission. In addition, significantly lower rates of moderate-severe S-STAI score were observed in the VR group before the procedure as compared to the routine management group. Changes in pulse and blood pressure were not statistically different, although a clinical reduction was noted in the systolic blood pressure in the VR group post-procedure.

Recent studies have explored the effectiveness of stress reduction techniques for patients undergoing medical procedures, particularly in the context of embryo transfer. Dviri et al. [11] implemented a VR intervention prior to embryo transfer, measuring outcomes such as clinical pregnancy rate and anxiety levels assessed via the STAI questionnaire and vital signs. Their preliminary findings indicated that VR exposure did not significantly reduce anxiety, and although there was a trend towards a higher clinical pregnancy rate in the intervention group, it was not statistically significant. Similarly, Di Guardo et al. [12] investigated the use of VR during embryo transfer, assessing anxiety through vital signs, the VAS scale, the STAI, and the Generalized Anxiety Disorder Questionnaire (GAD-7). While they observed decreased blood pressure values, the anxiety scores were comparable between the intervention and control groups, leading the authors to conclude that VR was not an effective tool for reducing anxiety during embryo transfer. In contrast to these studies, a recent [19] clinical trial in adult patients who were going through an elective surgical procedure requiring general anesthesia (38 men and 36 women) and tested the VR-based intervention versus no intervention demonstrated a significantly reduced pre-operative anxiety post VR exposure. Additional support arises from a pediatric systematic review and meta-analysis indicating that virtual reality is a useful tool to reduce pain and anxiety in a range of medical procedures, as it significantly decreases pain and anxiety outcomes when compared to the usual care [20]. These reports reflect an ongoing interest in stress reduction techniques for patients undergoing medical procedures.

While the observed reduction in anxiety levels was statistically significant, the magnitude of change in our population was relatively modest. Given the lack of a universally accepted threshold for clinical significance in STAI score reductions, the practical impact of this finding remains to be further explored. However, similar reductions have been reported as meaningful in previous studies on anxiety in reproductive medicine. For example, a randomized study on IVF patients undergoing a psychosocial intervention during counselling and treatment found that a 2-point reduction in state anxiety was statistically significant [21]. Additionally, another study reported a 4-point reduction as highly significant in the same patient population [22].

The strengths of our study include its prospective, randomized design, based on a power calculation and utilizing a well-known validated questionnaire. We evaluated a specific group of patients that has not been previously addressed in this aspect. This may account for the discrepancies observed between our results and those of prior studies involving reproductive procedures, specifically embryo transfer. We chose this population due to the immense popularity that planned oocyte cryopreservation has gained worldwide.

Mean stress levels in our patient cohort were lower than those reported in previous articles, and the study was limited to a specific population. Though promising, our results cannot necessarily be generalized, and further studies are required to validate our findings; our study was conducted in a specific demographic and clinical setting. Factors such as cultural differences in anxiety perception, variations in pain management protocols, and differing levels of familiarity with VR technology may influence outcomes in other patient groups. Future research in diverse populations is needed to determine the broader applicability of our findings. We acknowledge as a limitation of our study that healthcare professionals involved in egg retrieval were not blinded to patient allocation and that complete blinding was not feasible due to the nature of the VR device.

Despite this limitation, the questionnaires and research tools were provided by the daycare nurses, who were not involved in the fertility work-up/surgical procedure, and the questionnaires were completed directly by the patient, not the caregiver. This ensures that the responses reflect the patient’s own experience and perceptions. Additionally, the presence of a chaperone may influence patient anxiety at multiple points during the procedure and could have affected our results, despite the similar baseline anxiety levels between groups. Another limitation of our study is a lack of a long-term follow-up to assess whether the reduction in anxiety observed after VR exposure has any lasting impact beyond the immediate perioperative period. Future research should explore the durability of these effects and their potential influence on subsequent fertility treatments or patient decision-making.

To conclude, our results support exposing patients to VR prior to planned oocyte retrieval for cryopreservation as a tool to reduce the pre-procedure stress and anxiety. This approach is practical, requiring no additional staff training or complex implementation. While VR can be integrated into clinical settings worldwide, accessibility and cost may vary across institutions and healthcare systems, potentially limiting its widespread adoption. Further research is warranted to assess the effects of VR on patients’ satisfaction and overall perceptions of the procedure, as well as its feasibility in diverse clinical environments. Future interests involve using VR as a tool to reduce anesthesia doses in the broader IVF population and specifically among those undergoing planned oocyte cryopreservation.

## Data Availability

Data will be available upon request.
